# Association of sociodemographic and clinical factors with the quality of life of Brazilian individuals with Neurofibromatosis type 1: a cross-sectional study^☆^

**DOI:** 10.1016/j.abd.2023.08.011

**Published:** 2024-03-16

**Authors:** Natália Parenti Bicudo, Carla Maria Ramos Germano, Roberta Teixeira de Moraes, Lucimar Retto da Silva de Avó, Rosalie E. Ferner, Débora Gusmão Melo

**Affiliations:** a Department of Medicine, Universidade Federal de São Carlos, São Carlos, SP, Brazil; b National Neurofibromatosis Service, Department of Neurology, Guy's and St Thomas' NHS Foundation Trust, London, UK

**Keywords:** Brazil, Genetic disease, Neurofibromatosis type 1, Phenotype, Quality of life, Rare disease, Sociodemographic factors

## Abstract

**Background:**

Neurofibromatosis type 1 (NF1) is a rare genetic disorder with a wide range of clinical manifestations, notably neurocutaneous features, that can lead to emotional and physical consequences.

**Objectives:**

This study assessed the influence of sociodemographic factors and clinical features of the disease on the quality of life of Brazilian individuals with NF1.

**Methods:**

This is a descriptive cross-sectional study. Data were collected from 101 individuals with NF1 using the Brazilian version of the Impact of NF1 on Quality of Life Questionnaire (INF1-QoL), a form with information on sociodemographic characteristics, and an NF1 visibility self-evaluation scale. The relationship between variables was evaluated through statistical testing, and the significance level was defined as 0.05.

**Results:**

The study included 101 adults with NF1 aged 18 to 59 years, with a mean age of 35.54 years (±9.63) and a female predominance (n = 84, 83.17%). The mean total INF1-QoL score was 10.62 (±5.63), with a median of 10, minimum value of 0, and maximum of 31 points. Two characteristics of the participants were significantly associated with the quality of life: educational level (p = 0.003) and familial history of NF1 (p = 0.019). There was a statistically significant correlation between the INF1-QoL score and the degree of disease visibility (rho = 0.218; p = 0.028).

**Study limitations:**

Cross-sectional study, conducted with a convenience sample and using self-reported measures.

**Conclusions:**

The findings support the significant impact of NF1 on quality of life. The authors recommend multidisciplinary follow-up for patients, with adherence to anticipatory clinical care measures, adequate pain control, psychological assistance, and genetic counseling.

## Introduction

Neurofibromatosis Type 1 (NF1) is a rare genetic disorder involving multiple organ systems that is characterized by a wide range of clinical manifestations, most notably neurocutaneous features.^1^ The disease affects both sexes equally, and its prevalence has been estimated at 1 in 2,000 to 4,000 individuals.^2^

Common findings of NF1 include café-au-lait macules, melanocytic hamartomas in the iris known as Lish nodules, cutaneous neurofibromas, skinfold freckling, plexiform neurofibromas, optic pathway glioma, bone, endocrinological and neurological abnormalities, learning disabilities, and behavioral problems. Clinically, the phenotypic expression is highly variable.^3^ Almost all individuals have small benign cutaneous tumors that increase in number and become more visible with age. Approximately 30% of adults have visible plexiform neurofibromas, while 20% have internal plexiform neurofibromas detectable only by imaging exams.^4^

NF1 is a tumor suppressor gene that encodes a protein called neurofibromin, which regulates the RAS pathway. Neurofibromin is abundantly expressed in neurons, oligodendrocytes, and Schwann cells.^1^ Individuals with NF1 have a cumulative risk of malignancy of 20% to 39% when they are 50 years old, with a lifetime cancer risk of roughly 60%.^1^ The increased lifetime risk of developing cancer has prompted research into therapies that inhibit tumor growth.^3^

The visibility of skin diseases may lead to severe psychological distress and social burdens. In individuals with NF1, disease visibility is closely related to psychiatric morbidities, such as anxiety and depression, and impairs the quality of life.^4^, ^5^, ^6^, ^7^, ^8^ Disease severity and pain interfere with personal functionality and are also associated with impaired quality of life.^7^, ^8^, ^9^, ^10^, ^11^

In 2017, a group of researchers from the United Kingdom created the Impact of NF1 on Quality of Life Questionnaire (INF1-QoL), which is suitable as an assessment tool in the clinical setting as well as a patient-reported outcome measure in clinical trials and therapeutic interventions. It is a 14-question questionnaire with responses ranging from 0 to 3. The questionnaire has a maximum potential score of 42, with higher scores indicating a greater impact on quality of life. The original English version of the INF1-QoL is a validated, reliable, disease-specific questionnaire that correlates moderately with disease severity.^10^ The INF1-QoL questionnaire was adapted and validated in the Brazilian context and is available to professionals who assist these patients.^11^

This study aimed to assess the influence of sociodemographic factors and the visibility of the disease on the quality of life of Brazilian individuals with NF1 using the INF1-QoL questionnaire.

## Materials and methods

### Study design and ethical considerations

This is a descriptive cross-sectional study, developed after approval by the Committee for Ethical Compliance in Research Involving Human Beings at the Universidade Federal de São Carlos. All participants signed an Informed Consent Form before providing data. This study follows the recommendations from the Strengthening the Reporting of Observational Studies in Epidemiology (STROBE).^12^

### Sampling and data collection

The study was carried out with a convenience sample^13^ of 101 individuals with NF1 recruited through social networks over the Internet. Invitations to participate in the study were shared with Brazilian Facebook groups of people with NF1. The individuals’ inclusion criteria were: (1) being over 18 years old; (2) having a medical diagnosis of NF1; (3) being Brazilian; and (4) being accessible and interested in research participation.

Data collection was gathered through a self-report online form from October 2021 to January 2022. Data collection instruments were the Brazilian version of the INF1-QoL^11^ that is available at https://doi.org/10.6084/m9.figshare.19617162.v1, a questionnaire with information on sociodemographic features prepared for this study (Appendix S1, Supporting Information), and an NF1 visibility self-evaluation scale adapted from the Ablon scale.^14^ The Ablon scale is based on the appearance of a fully dressed person and how easily physical signs such as café-au-lait spots, tumors on the neck or face, or a noticeable limp, can be perceived in impersonal interactions.^14^ The authors developed and validated a scale for self-evaluation of NF1 visibility that consists of eight yes/no questions, allowing us to classify NF1 visibility as degree 1 (mild), degree 2 (moderate), or degree 3 (severe) according to the combination of each item response ^15^

### Data analysis

Descriptive analyses were performed for the personal and clinical characteristics of the participants and the INF1-QoL scores.

The INF1-QoL reliability was evaluated in terms of internal consistency using Cronbach’s alpha index, and values ≥ 0.70 were considered adequate.^16^

The normality of the quality of life variable, measured using the total INF1-QoL score, was verified using the Kolmogorov-Smirnov test with Lilliefors correction. Since the normality of the quality of life variable was rejected (D = 0.1169; p = 0.0017), non-parametric statistical methods were used.

The difference of the quality of life scores in relation to sociodemographic and clinical factors was analyzed using the Mann-Whitney or Kruskal-Wallis tests with Dunn's post-test, according to the number of groups in each variable.

Spearman correlation was used to verify the degree of binary correlation between the quality of life and the NF1 degree of visibility. The intensity of the correlation coefficient (rho) was established as a weak correlation between 0 and 0.3, a moderate correlation between > 0.3 and 0.6, and a strong correlation >0.6.

Statistical analyses were performed using the JASP 0.16.3.0 (https://jasp-stats.org)^17^ and a p-value of < 0.05 was considered statistically significant.

## Results

The participants were from 15 different states across the country, with a predominance of the states of São Paulo (n = 40; 39.60%), Minas Gerais (n = 12; 11.89%), and Paraná (n = 8; 7.92%). They ranged in age from 18 to 59 years old, with a mean age of 35.54 years (± 9.63), and they were mostly female (n = 84, 83.17%). Table 1 presents the participants’ sociodemographic information.

**Table 1:** Sociodemographic characteristics and visibility disease of individuals with NF1 (n = 101).

Features	n (%)
**Gender**	
Female	84 (83.17)
Male	17 (16.83)
**Age (years)**	
18–25	17 (16.83)
> 25–30	14 (13.86)
> 30–35	18 (17.82)
> 35–59	52 (51.49)
**Marital status**	
Single	60 (59.41)
Married or living together	39 (36.61)
Divorced	2 (1.98)
**Educational level**	
Incomplete primary education	2 (1.98)
Complete primary education	1 (1)
Incomplete secondary education	7 (6.93)
Complete secondary education	27 (26.73)
Vocational training course	7 (6.93)
Incomplete or complete higher education	40 (39.60)
Postgraduate education	17 (16.83)
**Number of children**	
None	61 (60.40)
1	20 (19.80)
2	12 (11.88)
≥3	8 (7.92)
**Family history of NF1**	
No	54 (53.47)
Yes, parents with NF1	19 (18.81)
Yes, children with NF1	19 (18.81)
Yes, other relatives with NF1	9 (8.91)
**NF1 visibility**	
Degree 1	28 (27.72)
Degree 2	20 (19.80)
Degree 3	53 (52.48)

**Figure 1:**
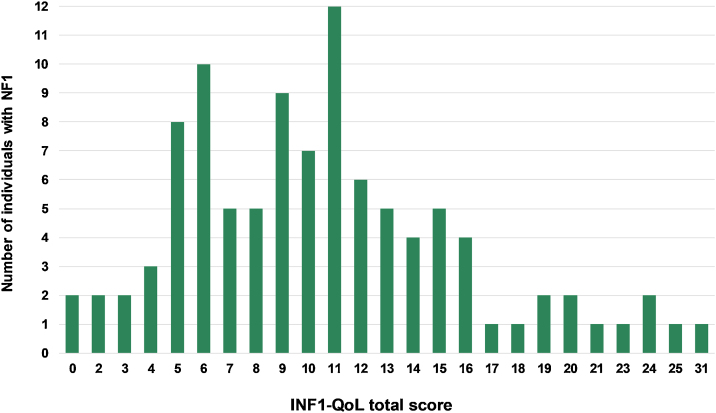
Total INF1-QoL score distribution in participants (n = 101).

**Figure 2:**
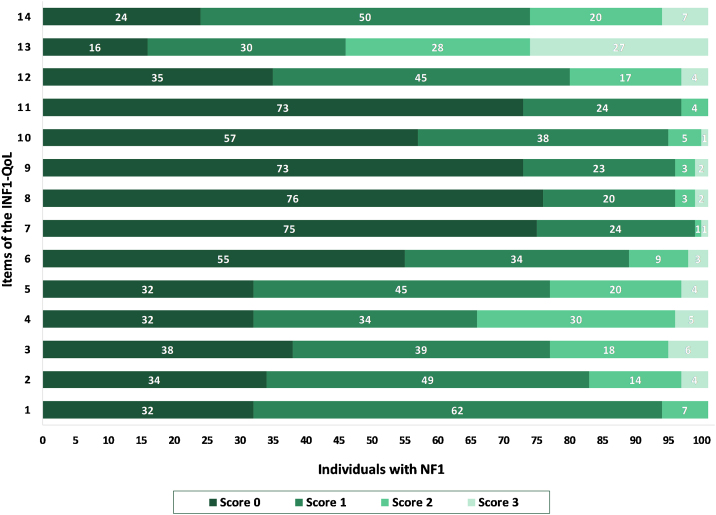
Frequency diagram of responses to the 14-items of the INF1-QoL among participants (n = 101).

The mean total INF1-QoL score was 10.62 (±5.63; 95% IC 9.51–11.73), with a median of 10 (95% IC 9.0–11.0), a minimum of 0, and a maximum of 31 points. Fig. 1 depicts the results of the total INF1-QoL score among the participants, while Fig. 2 presents the frequency diagram of responses to the 14 items of the INF1-QoL.

Cronbach’s alpha index for the total INF1-QoL was 0.8009, with values ranging from 0.7535 to 0.7963 for each questionnaire question (Table S1, Supporting Information).

Table 2 shows further relationships between sociodemographic and clinical variables and the INF1-QoL score. There was a significant difference in the mean total INF1-QoL score according to the educational level (p = 0.003). Regarding this, the “incomplete/complete primary education or incomplete secondary education” group differed from the “complete secondary education” (18.40 ± 6.57 vs. 10.07 ± 5.25, p < 0.001), “vocational training course” (18.40 ± 6.57 vs. 10.86 ± 3.44, p = 0.030), “incomplete/complete higher education” (18.40 ± 6.57 vs. 9.48 ± 4.62, p < 0.001) and “postgraduate education” (18.40 ± 6.57 vs. 9.53 ± 5.43, p < 0.001) groups.

**Table 2:** The INF1-QoL score distribution according to the sociodemographic and clinical variables (n = 101).

Variables	n	INF1-QoL score			p-value^a^
		Mean	SD	Median	
**Gender**					
Female	84	10.23	5.25	10	0.177
Male	17	12.59	7.08	12	
**Age (years)**					
18–25	17	9.06	5.36	10	0.471
> 25–30	14	9.43	4.65	7.5	
> 30–35	18	11.72	5.49	11	
> 35–59	51	11.08	5.97	10	
**Marital status**					
Married or living together	39	10	6.11	9	0.301
Single/Divorced	62	11.02	5.32	10.5	
**Educational level**					
Incomplete/complete primary education or incomplete secondary education	10	18.40	6.57	17	**0.003**^c^
Complete secondary education	27	10.07	5.25	10	
Vocational training course	7	10.86	3.44	12	
Incomplete/complete higher education	40	9.48	4.62	9	
Postgraduate education	17	9.53	5.43	8	
**Number of children**					
None	61	9.92	4.94	10	0.212
1	20	10.70	5.12	10	
2	12	14.42	7.43	12.5	
≥3	8	10.13	7.47	10	
**Familial history of NF1**					
No	54	11.67	5.61	11	**0.019**^b^
Yes	47	9.43	5.46	9	
**NF1 visibility**					
Degree 1	28	9.11	5.48	9	0.088
Degree 2	20	9.85	5.20	9	
Degree 3	53	11.72	5.72	11	

a Mann-Whitney or Kruskal-Wallis tests.

b p < 0.05.

c p < 0.01.

Furthermore, individuals with a positive family history of NF1 had lower INF1-QoL scores and, therefore, a lower impact on their quality of life (11.67 ± 5.61 vs. 9.43 ± 5.46, p = 0.019).

Although there was no significant difference in the mean INF1-QoL score according to the degree of disease visibility (p = 0.088), the INF1-QoL score was weakly correlated with the degree of disease visibility (rho = 0.218, p = 0.028), indicating that the more visible the disease, the worse the quality of life.

## Discussion

Regarding NF1 individuals’ quality of life, the INF1-QoL question that achieved the highest score was question number 13, which asked about the effect of NF1 on role and outlook on life (e.g., career, confidence, relationships, caring for family, having children, fear of passing on NF1 to children). These results are consistent with those of the original English questionnaire, where the highest score was also obtained for the same question.^10^ The authors believe that impairment in role and life perspective is closely related to feelings of uncertainty regarding the disease, as reported in previous qualitative research.^5^, ^6^, ^18^ There are two main dimensions in which the uncertainty typically associated with NF1 impacts those living with the condition. The first dimension is concerned with disease progression. This has already been mentioned among adults of all ages and degrees of severity, and often the anxiety about what may happen in the future is greater than the present symptoms of the disorder. The most concerning aspect is the possibility of neurofibromas growing in size or number, as well as the chance of developing cancer. A second dimension is related to family planning. Anxiety and uncertainty about hereditary diseases, such as NF1, can lead to a behavior interpreted as “genetic responsibility”,^18^ in which individuals sometimes decide not to have children in order to avoid perpetrating the genetic condition in future generations.^5^, ^6^, ^18^ These issues might be related to the fact that more than half of the sample in the present study was comprised of single individuals.

The second highest-scoring INF1-QoL question was 14, which addressed depression and anxiety, followed by questions 3 and 4, which addressed physical pain. These findings are consistent with existing studies in the literature, which showed that physical pain, anxiety, and depression were the main affected factors in the lives of those with the disorder^8^, ^19^, ^20^ and suggested screening for pain and depression in routine assessments of patients with NF1.^8^

The etiology of NF1 pain is not totally known. Studies of NF1-rat models highlighted CRMP2 (Collapsin Response Mediator Protein 2), an intracellular phosphoprotein predominantly expressed in the nervous system during development and involved in axon guidance and growth, as a key player in the development of NF1 pain.^21^ Current treatments specifically targeting NF1-pain are scarce, and most of the time patients are managed with general over-the-counter medication and surgery for removal of painful neurofibromas, neither of which result in complete attenuation of NF1-pain. Additionally, pain is a subjective symptom, and a biopsychosocial approach can provide the most benefit in relieving the complaint.^22^ It’s worth mentioning that studies on gender-based differences involved in NF1 pain are needed since Crawford et al. (2015) demonstrated a greater effect of NF1 pain on mood in women with NF1.^5^ Individuals with NF1 pain have barriers to obtaining comprehensive pain-specific treatment, including difficulty accessing medical services and the limited availability of trained professionals with disease-specific knowledge.^23^ Adequate pain control is fundamental, as unrelieved pain generates psychological and emotional distress in addition to impairing sleep, social activities, and cognitive functions.^8^, ^22^ Understanding pain in the context of NF1 will upgrade clinical care and improve the quality of life of NF1 patients.

The previously predicted relationship between visibility and quality of life^4^, ^5^, ^6^, ^7^, ^8^ was confirmed in the present study. The majority of participants identified themselves as degree 3 on the visibility self-evaluation scale, most likely because those with more severe clinical conditions were more interested in engaging in research and understanding their condition. Although the distribution of the mean quality of life score did not differ substantially between patients with disease visibility degrees of 1, 2, or 3, the authors found a trend toward an association between increased NF1 visibility and worse quality of life. In the British study, INFI-QoL scores correlated moderately with clinical disease severity.^10^

The positive association revealed in these results between schooling and quality of life was already expected. The consequences of education on the quality of life are complex and span numerous areas and dimensions of life. According to Edgerton et al. (2012), there are three main implicit ways in which education can improve a person's quality of life: (a) acquiring knowledge and analytical skills that can be used to direct individuals' behavior; (b) changing individuals' preferences in ways that allow them to reorient their general values or priorities; and (c) reducing constraints and creating opportunities.^24^ It is worth mentioning that the authors cannot estimate the contribution of problems with learning, which are often in NF1, to difficulty filling out the research form, which may be related to the small participation of individuals with primary education in this study.

Family history was unexpectedly linked to the INF1-QoL scores. When a disease is hereditary, family experiences are passed down across generations.^25^, ^26^ The authors believe that it may be easier to understand and accept NF1 symptoms when other family members are affected by the same condition. Furthermore, greater awareness regarding health care and legal rights relating to the disorder may occur, which may reduce some practical life challenges for those affected.

This study has some limitations. First, it is a cross-sectional study and therefore does not address a cause-and-effect relationship. A longitudinal study with follow-up would help to clarify the nature of the associations found in this study. Second, the data collection was performed using self-reported measures. Study participants were recruited from individuals who had received a medical diagnosis of NF1 and who, as a result, were participating in virtual communities about the condition. The authors relied on participants' information about their condition. Furthermore, all participants filled out the NF1 visibility self-evaluation scale, which allowed classifying them concerning the degree of NF1 visibility and confirmed that all had café-au-lait spots. Thus, the research was conducted with a convenience sample, undoubtedly biased, representing people with access to the Internet and social networks, which makes it difficult to generalize these study results. There was a predominance of female participants, maybe because they were more likely to fill out the form.

## Conclusion

The results of this study corroborate the significant impact of NF1 on quality of life. For comprehensive health care for affected individuals, the authors suggest a multidisciplinary team follow-up with adherence to anticipatory clinical care measures to identify the disease's main complications early and offer timely treatment. Adequate pain control is fundamental, as unrelieved pain generates anxiety and physical and emotional stress, in addition to impairing sleep, social activities, and cognitive functions.

Furthermore, genetic counseling may help in adjusting roles and life perspectives in addition to supporting decisions about the reproductive future. Healthcare providers should be aware of the possible impact of the disorder on psychological distress and social burdens, and psychological assistance should be provided for patients whenever indicated.

## Financial support

This study was financed in part by the Coordenação de Aperfeiçoamento de Pessoal de Nível Superior ‒ Brasil (CAPES) ‒ Finance Code 001.

## Authors’ contributions

Natália P. Bicudo: Conception and planning of the study; collection, analysis, and interpretation of data; statistical analysis; drafting and editing of the manuscript; critical review of the manuscript; approval of the final version of the manuscript.

Carla M. R. Germano: Analysis and interpretation of data; critical review of the manuscript; approval of the final version of the manuscript.

Roberta T. de Moraes: Analysis and interpretation of data; critical review of the manuscript; approval of the final version of the manuscript.

Lucimar R. S. de Avó: Analysis and interpretation of data; critical review of the manuscript; approval of the final version of the manuscript.

Rosalie E. Ferner: Analysis and interpretation of data; critical review of the manuscript; approval of the final version of the manuscript.

Débora G. Melo: Conception, planning, and supervision of the study; analysis and interpretation of data; statistical analysis; editing of the manuscript; critical review of the manuscript; approval of the final version of the manuscript.

## Conflicts of interest

None declared.
