# Supplementary material for: Association of sociodemographic and clinical factors with the quality of life of Brazilian individuals with Neurofibromatosis type 1: a cross-sectional study
Source: An Bras Dermatol. 2024 Mar 16;99(4):520–6. doi: 10.1016/j.abd.2023.08.011 (PMC11220916; doi:10.1016/j.abd.2023.08.011)
Supplement: Supplementary file 1 [file mmc1.docx]

ABD-D-23-00373_ Supplementary material

**Table S1** Cronbach's alpha index of the total INF1-QoL and its 14 items (n=101).

| **INF1-QoL** | **Cronbach's alpha index** |
| --- | --- |
| 1 | 0.7963 |
| 2 | 0.7772 |
| 3 | 0.7535 |
| 4 | 0.7605 |
| 5 | 0.7744 |
| 6 | 0.7738 |
| 7 | 0.7792 |
| 8 | 0.7754 |
| 9 | 0.7761 |
| 10 | 0.7786 |
| 11 | 0.7745 |
| 12 | 0.7566 |
| 13 | 0.7736 |
| 14 | 0.7547 |
| Total | 0.8009 |

**Appendix S1** Sociodemographic profile form.

| **Information about you:** | | | | | | | | |
| --- | --- | --- | --- | --- | --- | --- | --- | --- |
| 1) Age: ___________________ | | | | | | | | |
|  | | | | | | | | |
| 2) Gender: | | | | | | | | |
| ( ) Female | | | | | ( ) Male | | | |
|  | | | | | | | | |
| 3) Date of birth: ________________ | | | | | | | | |
|  | | | | | | | | |
| 4) State where you live (residence): ___________________ | | | | | | | | |
|  | | | | | | | | |
| 5) Marital status: | | | | | | | | |
| ( ) Married or living together | | ( ) Single | | | | ( ) Divorced | | ( ) Widowed |
| 6) Educational level: | | | | | | | | |
| ( ) Incomplete primary education | | | | ( ) Complete primary education | | | | |
| ( ) Incomplete secondary education | | | | ( ) Complete secondary education | | | | |
| ( ) Vocational training course | | | | ( ) Incomplete higher education | | | | |
| ( ) Complete higher education | | | | ( ) Postgraduate education | | | | |
| 7) Family history of NF1 | | | | | | | | |
| ( ) Yes | | | | ( ) No | | | | |
| If yes, who else in the family has NF? | | | | | | | | |
| ( ) Parents (mother or father) | | | ( ) Children | | | ( ) Others. Describe: _____________ | | |
| 8) Number of children | | | | | | | | |
| ( ) None | ( ) 1 child | | | | ( ) 2 children | | ( ) 3 or more children | |
